# Daytime sleepiness in Japanese patients with multiple system atrophy: prevalence and determinants

**DOI:** 10.1186/1471-2377-12-130

**Published:** 2012-11-01

**Authors:** Takayoshi Shimohata, Hideaki Nakayama, Masahiko Tomita, Tetsutaro Ozawa, Masatoyo Nishizawa

**Affiliations:** 1 Department of Neurology, Brain Research Institute, Niigata University, Niigata, Japan; 2 Division of Respiratory Medicine, Niigata University Graduate School of Medical and Dental Sciences, Niigata, Japan; 3 Department of Otolaryngology, Niigata University Graduate School of Medical and Dental Sciences, Niigata, Japan

**Keywords:** Multiple system atrophy, Excessive daytime sleepiness, Epworth Sleepiness Scale, Sleep-disordered breathing, Abnormal periodic leg movements in sleep

## Abstract

**Background:**

The recent SLEEMSA study that evaluated excessive daytime sleepiness (EDS) in Caucasian patients with multiple system atrophy (MSA) demonstrated that EDS was more frequent in patients (28%) than in healthy subjects (2%). However, the prevalence and determinants of EDS in other ethnic populations have not been reported to date.

**Methods:**

We performed a single-hospital prospective study on patients with probable MSA. To ascertain the prevalence and determinants of EDS in Japanese MSA patients, we assessed the patients’ degree of daytime sleepiness by using the Japanese version of the Epworth Sleepiness Scale (ESS). In addition, we investigated the effects of sleep-disordered breathing (SDB) and abnormal periodic leg movements in sleep (PLMS), which were measured by polysomnography, on the patients’ ESS scores.

**Results:**

A total of 25 patients with probable MSA (21 patients with cerebellar MSA and 4 patients with parkinsonian MSA) were included in this study. All patients underwent standard polysomnography. The mean ESS score was 6.2 ± 0.9, and EDS was identified in 24% of the patients. SDB and abnormal PLMS were identified in 24 (96%) and 11 (44%) patients, respectively. The prevalences of EDS in patients with SDB and abnormal PLMS were 25% and 18%, respectively. No correlations were observed between ESS scores and the parameters of SDB or abnormal PLMS.

**Conclusions:**

The frequency of EDS in Japanese patients with MSA was similar to that in Caucasian MSA patients. SDB and abnormal PLMS were frequently observed in MSA patients, although the severities of these factors were not correlated with EDS. Further investigations using objective sleep tests need to be performed.

## Background

Excessive daytime sleepiness (EDS) is believed to be caused by disturbed or inadequate sleep [1]. EDS is common in older adults [2] and is more frequently observed in patients with underlying sleep disorders, such as sleep apnea syndrome (SAS), abnormal periodic leg movements in sleep (PLMS), restless legs syndrome (RLS), as well as neurodegenerative disorders such as Parkinson’s disease (PD) and Alzheimer’s disease [3].

In addition, EDS has been considered a common manifestation of multiple system atrophy (MSA) [3,4]. MSA patients can develop sleep-disordered breathing (SDB), which is associated with upper-airway obstruction or central respiratory dysfunction [5-7]. Furthermore, patients with MSA are known to show impairment of the neural systems involved in the maintenance of the waking state, including the cholinergic neurons of the mesopontine tegmentum [8], serotonergic neurons of the rostral raphe [9], hypocretin/orexin neurons of the lateral hypothalamus [10], and putative wake-active dopaminergic neurons in the ventral periaqueductal gray matter [11]. These clinical and pathological findings suggest an increase in sleep disturbances and subsequent EDS in MSA patients. In fact, the recent SLEEMSA study that evaluated 86 Caucasian MSA patients (73 parkinsonism and 13 cerebellar) demonstrated that EDS was present in 28% of patients with MSA, which was more frequent than that in healthy subjects (2%) [12]. This study also demonstrated that the symptoms of SDB, which were indicated by the presence of snoring or difficulties in breathing during sleep, predicted EDS in MSA patients. However, as far as we know, there have been no studies that have investigated the prevalence of EDS in other ethnic populations and the effects of SDB or abnormal PLMS [13], as measured by polysomnography (PSG), on EDS in MSA patients.

In the present study, we aimed to ascertain the prevalence of EDS in Japanese MSA patients by using the Epworth Sleepiness Scale (ESS) [14]. In addition, we investigated the effects of SDB and abnormal PLMS, which were measured by PSG, on the ESS scores.

## Methods

### Subjects

This study was approved by the Ethics Committee of the Niigata University School of Medicine. We performed a single-hospital prospective study on patients with probable MSA [15] who were admitted to our hospital between 2005 and 2011. Written informed consent was obtained from all participants. A patient’s degree of daytime sleepiness was assessed using the Japanese version of the ESS [16]. Possible scores ranged from 0 for the lowest degree of sleepiness and 24 for the highest degree of sleepiness. EDS was defined when the ESS score was greater than 10 [17]. Patients who had not experienced the situations included in the questionnaire because of disease progression were asked to estimate their answers. Assessments were also performed with the Unified Multiple System Atrophy Rating Scale (UMSARS) [18] and cognitive function tests, including the Mini-Mental State Examination (MMSE) [19] and Frontal Assessment Battery (FAB) [20]. All patients underwent standard PSG.

### Data acquisition

Standard PSG was performed with the following montages: electroencephalography (C3-A2, C4-A1, O1-A2, and O2-A1); electrooculography; submental electromyography; a nasal cannula to measure nasal pressure and a thermistor to monitor nasal and oral flow; anterior tibialis movement sensors; inductive plethysmography for thoracoabdominal motion; electrocardiography; and arterial oxygen saturation. Esophageal pressure was also measured in order to monitor the respiratory effort. Signals were continuously recorded on a 24-channel polygraph (SomnoStarPro; VIASYS, Yorba Linda, CA, USA), and the PSG records were evaluated by clinical technologists who were certified by the Japanese Society of Sleep Research and blinded to the patients’ clinical status. The scoring of sleep stages, arousals, and respiratory events was done manually from the PSG records according to the 1999 recommendations of the American Academy of Sleep Medicine Task Force [21]. SDB was defined as an apnea-hypopnea index (AHI) of 5 or more apnea or hypopnea episodes/h, and severe SDB was defined as an AHI of 30 or more apnea or hypopnea episodes/h. The periodic leg movement (PLM) index was calculated on the basis of the number of PLMs/h in sleep. A PLM index greater than 15 was defined as an abnormal PLMS according to Version 2 of the International Classification of Sleep Disorders. The PLM arousal index was defined as the number of PLM-related electroencephalographic arousals/h of sleep. RLS was diagnosed when patients met the 4 minimal criteria of the syndrome as defined by an international study group [22]. In the event of an inability to fall asleep, subjects were administered a nonbenzodiazepine sedative/hypnotic (zolpidem, 5 mg) at the beginning of polysomnography. The calculation of the L-3,4-dihydroxyphenylalanine (L-dopa)-equivalent dose (LED) was based on its theoretical equivalence to L-dopa, as described in previous reports [23-25] as follows: 100 mg L-dopa with dopa-decarboxylase inhibitor = 1 mg pergolide = 1.5 mg of cabergoline = 1 mg of pramipexole. Fiberoptic laryngoscopy was performed during wakefulness as well as under sedation by intravenously injecting propofol [6].

### Statistical analyses

Relationships between the ESS scores and variables related to clinical and sleep characteristics were analyzed by a Spearman's rank correlation test. Unpaired Student’s *t*-tests were used to compare the quantitative variables, and a Mann–Whitney rank sum test was used when appropriate. Statistical analyses were performed using SigmaPlot version 11.0 with the level of significance set at P < 0.05. The data are presented as mean ± standard error of mean.

## Results

### Clinical characteristics

We recruited 25 consecutive patients with probable MSA (Table 1). Of the 25 MSA patients, 21 (84%) had MSA-cerebellar (MSA-C), and the remaining 4 (16%) had MSA-parkinsonism (MSA-P). This subtype frequency is consistent with the results obtained by a recent national epidemiological study in Japan, which showed that 83% of MSA patients (n = 3,341) have MSA-C, suggesting that the MSA-C subtype is more frequent in Japanese patients than in western patients [26].

**Table 1:** Clinical and sleep characteristics of multiple system atrophy (MSA) patients

**Variables**	**MSA patients**
Patients	25
Male:Female	10:15
Age at onset (years)	62.3 ± 1.7 (46–78)
Disease duration (months)	45.9 ± 4.7 (12–96)
UMSARS	48.0 ± 4.0 (17–82)
ESS	6.2 ± 0.9 (0–15)
Total sleep time (min)	322 ± 18 (103–575)
Sleep efficiency (%)	61.9 ± 2.7 (35–95)
Stage N1 (%)	32.1 ± 2.26 (12.6–57.3)
Stage N2 (%)	46.3 ± 3.1 (11.4–69.8)
Stage N3 (%)	5.2 ± 1.4 (0–21.7)
Stage REM (%)	15.6 ± 2.5 (0–44.9)
Arousal index (/h)	43.0 ± 4.1 (0–79.9)
AI (/h)	19.7 ± 4.7 (0–80.8)
AHI (/h)	41.9 ± 6.4 (4.1–117.3)
Mean SpO_2_ (%)	93.1 ± 3.2 (82–97)
PLM index (/h)	44.2 ± 11.7 (0–166.0)
PLM arousal index (/h)	2.3 ± 0.9 (0–16.2)
LED (mg/day)	352 ± 50 (25–683)

Values are means ± standard error of the means (SEM; range). MSA, multiple system atrophy; UMSARS, unified multiple system atrophy rating scale; ESS, Epworth sleepiness scale; REM, rapid eye movement; AI, apnea index; AHI, apnea-hypopnea index; SpO_2_, oxygen saturation; PLM, periodic leg movement; LED, L-dopa-equivalent dose.

Four patients were able to walk without help, 20 were able to walk with the help of a walker, and 1 could not walk. All 25 patients showed autonomic failure: 15 patients had orthostatic hypotension [15], and 22 had urinary incontinence. Laryngeal stridor was present in 8 patients (32%). MMSE and FAB scores were 26.4 ± 0.6 (range, 17–30) and 13.7 ± 0.6 (range, 8–18), respectively. Eleven patients were treated with antiparkinsonian drugs: l-dopa without dopamine agonists (DAs) was administered to 8 patients, l-dopa and DAs (cabergoline or pergolide) were administered to 2 patients. One patient was administered pramipexole without l-dopa. The LED of the 11 patients was 345 ± 47 mg/day (range, 25–608 mg/day). Taltirelin hydrate (10 mg/day), which is a thyrotropin-releasing hormone analog used in Japan for the treatment of spinocerebellar degeneration, was administered to 10 patients. Antidepressants were administered to 4 patients. Clonazepam and antipsychotic drugs were not routinely administered to any of the patients. Ten patients received zolpidem (5 mg) at the beginning of their study because of difficulty in sleeping.

### Sleep characteristics

In MSA patients, the ESS score was 6.2 ± 0.9 (range, 0–15), and the prevalence of EDS was 24% (6/25) (Table 1). No MSA patients reported a sudden onset of sleep. PSG findings showed that the sleep efficiency of MSA patients was reduced (61.9 ± 2.7%). The sleep pattern of MSA patients was characterized by reductions in non-rapid eye movement (REM) stage 3 (N3; 5.2 ± 1.4%; normal value, 13–23%) and mild reductions in the REM stage (15.6 ± 2.5%; normal value, 20–25%) in comparison to the previously published normal values [27]. The AHI was 41.9 ± 6.4 (4.1–117.3) events per hour, and the central, mixed, and obstructive AHIs were 3.0 ± 1.6 (0–40.7), 2.5 ± 1.2 (0–23.3), and 36.4 ± 5.3 (3.4–82.5) events per hour, respectively. A total of 24 MSA patients (96%) fulfilled the SDB criteria, and the prevalence of EDS in MSA patients with SDB was 25% (6 out of the 24 patients). All patients had predominantly obstructive SDB, although 19 patients also had central SDH. The frequencies of vocal cord abductor paralysis, floppy epiglottis [28], and oropharyngeal obstruction were 15/25 (60%), 9/25 (36%), and 8/25 (32%), respectively. Eight patients developed upper airway obstructions at more than one site. Fourteen patients (56%) met the criteria for severe SDB, and the prevalence of EDS in the patients with severe SDB was 21% (3 out of the 14 patients). Abnormal PLMS was observed in 44% (11 of the 25 patients) of the patients, and the prevalence of EDS in the patients with abnormal PLMS was 18% (2 of the 11 patients). RLS was observed in 12% (3 out of the 25 patients) of the patients, and none of the patients with RLS showed EDS.

### Effects of clinical and sleep characteristics on ESS scores

No correlations were observed between the ESS scores and disease duration, disease severity as assessed by UMSARS scores, or cognitive function as assessed by MMSE and FAB scores (Table 2). The PSG findings indicated no correlations between ESS scores and the parameters of SDB [apnea index (AI), AHI, and mean oxygen saturation (SpO_2_) levels] and abnormal PLMS (PLM index and PLM arousal index). In the 11 patients treated with antiparkinson drugs, a significant positive correlation was found between ESS scores and LED (r = 0.662, *P* = 0.027).

**Table 2:** Correlations between the Epworth sleepiness scale (ESS) scores and clinical and sleep characteristics of multiple system atrophy (MSA) patients

**Variables**	***r *value**	***P *value**
Disease duration (months)	−0.063	0.766
UMSARS	0.210	0.337
MMSE	−0.047	0.825
FAB	−0.098	0.657
Total sleep time (min)	0.262	0.215
Sleep efficiency (%)	0.297	0.149
Stage N1 (%)	0.138	0.512
Stage N2 (%)	0.179	0.391
Stage N3 (%)	−0.267	0.197
Stage REM (%)	−0.311	0.131
Arousal index (/h)	−0.070	0.738
AI (/h)	0.069	0.744
AHI (/h)	0.098	0.640
Mean SpO_2_ (%)	0.080	0.705
PLM index (/h)	−0.218	0.296
PLM arousal index (/h)	0.066	0.755
LED (mg)	0.662	**0.027**

UMSARS, unified multiple system atrophy rating scale; MMSE, mini-mental state examination; FAB, frontal assessment battery; REM, rapid eye movement; AI, apnea index; AHI, apnea-hypopnea index; SpO_2_, oxygen saturation; PLM, periodic leg movement; LED, L-dopa-equivalent dose.

## Discussion

The present study showed that the ESS score in our MSA patients was 6.2 ± 0.9. This ESS score was higher than the previously published score in Japanese healthy subjects (4.1 ± 0.3; n = 79; mean age, 67.0 ± 1.2) [29]. The prevalence of EDS in our MSA patients was 24%. This prevalence was similar to that reported in the SLEEMSA study [12], which demonstrated that the prevalence of EDS in MSA patients (28%) was comparable to that in PD patients (29%), but the prevalence was higher than that in healthy subjects (2%). These findings raise the possibility that EDS is a common clinical feature in MSA patients despite the differences in ethnicity as well as in subtype [26,30].

We also analyzed the effects of SDB and abnormal PLMS on EDS. Consistent with the results of previous studies [6,13], SDB was frequently observed in our study. A total of 24 patients (96%) met the criteria for SDB, and 56% of the patients met the criteria for severe SDB. The recent SLEEMSA study demonstrated the correlation between SDB, which was based on questionnaires, and EDS [12]. However, this study showed that there was no correlation between ESS scores and the parameters of SDB, which are AI, AHI, and mean SpO_2_ levels, in MSA patients, whereas SDB scores correlated with the EDS in patients with obstructive SAS in other studies [14,31]. These results suggest that SDB may not necessarily have a strong influence on EDS in MSA patients.

Although abnormal PLMS may be observed in MSA patients, there is no evidence that an increased prevalence of abnormal PLMS causes sleep disturbances and EDS in MSA patients. Vetrugno et al. reported that 15 of 18 patients with MSA (88%) presented with abnormal PLMS [13]. In the present study, the prevalence of abnormal PLMS was 44%, indicating that abnormal PLMS was frequently associated with MSA despite the differences in ethnicity, although it was less frequent than in the previous study [13]. We speculate that abnormal PLMS may not have a strong influence on EDS in MSA patients, because we did not find any significant correlations between the ESS scores and the indexes for PLM and PLM arousal. A lack of correlation between EDS and AHI or PLMS suggested an impairment of the normal sympathetic response to sleep apnea in MSA.

We showed that the disease duration and severity, impairments of cognitive or frontal function, as well as sleep quality that was evaluated by PSG, were not associated with EDS, while we identified a dose-dependent effect of antiparkinson drugs on EDS. Although this finding was not consistent with the SLEEMSA study, which showed a lack of correlation between the amount of dopaminergic treatment and EDS [12], it suggests still another possibility that antiparkinson drugs may cause EDS, as is the case with PD [32]. However, because 84% of the patients in the present study had MSA-C, we were able to recruit only 11 patients who had been treated with antiparkinson drugs. In addition, it remains the possibility that there may be confounding factors that are correlated with EDS and LED, such as the disease severity and duration. Further studies should be performed in order to further investigate the effects of antiparkinson drugs on sleepiness in MSA patients.

As mentioned above, the major determinant of EDS in MSA patients remains unknown. It is possible that the involvement of the arousal system may be a determinant of EDS in patients with MSA. In addition, as has been reported for patients with Parkinson’s disease, several factors, such as depressive symptoms, hallucinations, psychosis, nocturia, and nocturnal motor symptoms, can be possible causes of the EDS in MSA patients [32].

This study had several limitations. The first limitation of this study was that some of the subjects were administered a DA (3 patients) or an antidepressant (4 patients), and these medications can cause daytime sleepiness. The second limitation was the uncertainty over whether ESS is a suitable method for evaluating daytime sleepiness in MSA patients. This uncertainty may stem from the difficulty in ascertaining that MSA patients accurately rated their likelihood of falling asleep in some situations that they did not experience after disease progression. These limitations suggest the need for further investigations to determine objective sleep tests, such as a multiple sleep latency test, in MSA patients.

## Conclusions

The frequency of EDS in Japanese MSA patients (24%) was similar to that in Caucasian MSA patients (28%) in spite of the difference in the frequency of subtypes. SDB and abnormal PLMS were frequently observed in MSA patients (96% and 64%, respectively), although the severities of these factors were not correlated with EDS.

## Abbreviations

AHI: Apnea-hypopnea index; DA: Dopamine agonist; EDS: Excessive daytime sleepiness; ESS: the Epworth Sleepiness Scale; FAB: Frontal Assessment Battery; LED: l-3,4-dihydroxyphenylalanine (l-dopa)-equivalent dose; MMSE: the Mini-Mental State Examination; MSA: Multiple system atrophy; MSA-C: MSA-cerebellar; MSA-P: MSA-parkinsonism; PD: Parkinson’s disease; PLM: Periodic leg movement; PLMS: Abnormal periodic leg movements in sleep; PSG: Polysomnography; REM: Rapid eye movement; RLS: Restless legs syndrome; SAS: Sleep apnea syndrome; SDB: Sleep-disordered breathing; UMSARS: the Unified Multiple System Atrophy Rating Scale.

## Competing interests

The authors declare that they have no conflicts of interest.

## Authors' contributions

TS drafted the first manuscript and contributed to the acquisition of data. HN, MT, and TO contributed to the acquisition and interpretation of the data. MN revised the manuscript that led to the final approval of the current submission. All authors read and approved the final manuscript.

## Pre-publication history

The pre-publication history for this paper can be accessed here:

http://www.biomedcentral.com/1471-2377/12/130/prepub

## References

[B1] Roehrs T Carskacon MA Dement WC Roth T Kryger MH, Roth T, Dement WC Daytime sleepiness and alertness Principles and Practice of Sleep Medicine 2000 3d W.B. Saunders, Philadelphia 43 52

[B2] Roberts RE Shema SJ Kaplan GA Strawbridge WJ Sleep complaints and depression in an aging cohort: A prospective perspective Am J Psychiatry 2000 157 1 81 88 10618017 10.1176/ajp.157.1.81

[B3] El-Ad B Korczyn AD Disorders of excessive daytime sleepiness–an update J Neurol Sci 1998 153 2 192 202 10.1016/S0022-510X(97)00291-8 9511878

[B4] Arnulf I Excessive daytime sleepiness in parkinsonism Sleep Med Rev 2005 9 3 185 200 10.1016/j.smrv.2005.01.001 15893249

[B5] Chokroverty S Sachdeo R Masdeu J Autonomic dysfunction and sleep apnea in olivopontocerebellar degeneration Arch Neurol 1984 41 9 926 931 10.1001/archneur.1984.04050200032014 6383299

[B6] Shimohata T Shinoda H Nakayama H Ozawa T Terajima K Yoshizawa H Matsuzawa Y Onodera O Naruse S Tanaka K Daytime hypoxemia, sleep-disordered breathing, and laryngopharyngeal findings in multiple system atrophy Arch Neurol 2007 64 6 856 861 10.1001/archneur.64.6.856 17562934

[B7] Silber MH Levine S Stridor and death in multiple system atrophy Mov Disord 2000 15 4 699 704 10.1002/1531-8257(200007)15:4<699::AID-MDS1015>3.0.CO;2-L 10928581

[B8] Schmeichel AM Buchhalter LC Low PA Parisi JE Boeve BW Sandroni P Benarroch EE Mesopontine cholinergic neuron involvement in Lewy body dementia and multiple system atrophy Neurology 2008 70 5 368 373 10.1212/01.wnl.0000298691.71637.96 18227417

[B9] Benarroch EE Schmeichel AM Sandroni P Parisi JE Low PA Rostral raphe involvement in Lewy body dementia and multiple system atrophy Acta Neuropathol 2007 114 3 213 220 10.1007/s00401-007-0260-3 17639427

[B10] Benarroch EE Schmeichel AM Sandroni P Low PA Parisi JE Involvement of hypocretin neurons in multiple system atrophy Acta Neuropathol 2007 113 1 75 80 17089135 10.1007/s00401-006-0150-0

[B11] Benarroch EE Schmeichel AM Dugger BN Sandroni P Parisi JE Low PA Dopamine cell loss in the periaqueductal gray in multiple system atrophy and Lewy body dementia Neurology 2009 73 2 106 112 10.1212/WNL.0b013e3181ad53e7 19597132 PMC2713188

[B12] Moreno-Lopez C Santamaria J Salamero M Del Sorbo F Albanese A Pellecchia MT Barone P Overeem S Bloem B Aarden W Excessive daytime sleepiness in multiple system atrophy (SLEEMSA study) Arch Neurol 2011 68 2 223 230 10.1001/archneurol.2010.359 21320989

[B13] Vetrugno R Provini F Cortelli P Plazzi G Lotti EM Pierangeli G Canali C Montagna P Sleep disorders in multiple system atrophy: a correlative video-polysomnographic study Sleep Med 2004 5 1 21 30 10.1016/j.sleep.2003.07.002 14725823

[B14] Johns MW A new method for measuring daytime sleepiness: the Epworth sleepiness scale Sleep 1991 14 6 540 545 1798888 10.1093/sleep/14.6.540

[B15] Gilman S Low PA Quinn N Albanese A Ben-Shlomo Y Fowler CJ Kaufmann H Klockgether T Lang AE Lantos PL Consensus statement on the diagnosis of multiple system atrophy J Neurol Sci 1999 163 1 94 98 10.1016/S0022-510X(98)00304-9 10223419

[B16] Takegami M Suzukamo Y Wakita T Noguchi H Chin K Kadotani H Inoue Y Oka Y Nakamura T Green J Development of a Japanese version of the Epworth Sleepiness Scale (JESS) based on item response theory Sleep Med 2009 10 5 556 565 10.1016/j.sleep.2008.04.015 18824408

[B17] Johns MW Sensitivity and specificity of the multiple sleep latency test (MSLT), the maintenance of wakefulness test and the epworth sleepiness scale: failure of the MSLT as a gold standard J Sleep Res 2000 9 1 5 11 10.1046/j.1365-2869.2000.00177.x 10733683

[B18] Wenning GK Tison F Seppi K Sampaio C Diem A Yekhlef F Ghorayeb I Ory F Galitzky M Scaravilli T Development and validation of the Unified Multiple System Atrophy Rating Scale (UMSARS) Mov Disord 2004 19 12 1391 1402 10.1002/mds.20255 15452868

[B19] Folstein MF Folstein SE McHugh PR “Mini-mental state”. A practical method for grading the cognitive state of patients for the clinician J Psychiatr Res 1975 12 3 189 198 10.1016/0022-3956(75)90026-6 1202204

[B20] Kugo A Terada S Ata T Ido Y Kado Y Ishihara T Hikiji M Fujisawa Y Sasaki K Kuroda S Japanese version of the Frontal Assessment Battery for dementia Psychiatry Res 2007 153 1 69 75 10.1016/j.psychres.2006.04.004 17599465

[B21] Sleep-related breathing disorders in adults: recommendations for syndrome definition and measurement techniques in clinical research. The Report of an American Academy of Sleep Medicine Task Force Sleep 1999 22 5 667 689 10450601

[B22] Allen RP Picchietti D Hening WA Trenkwalder C Walters AS Montplaisir J Restless legs syndrome: diagnostic criteria, special considerations, and epidemiology. A report from the restless legs syndrome diagnosis and epidemiology workshop at the National Institutes of Health Sleep Med 2003 4 2 101 119 10.1016/S1389-9457(03)00010-8 14592341

[B23] Goetz CG Blasucci L Stebbins GT Switching dopamine agonists in advanced Parkinson's disease: is rapid titration preferable to slow? Neurology 1999 52 6 1227 1229 10.1212/WNL.52.6.1227 10214748

[B24] Inzelberg R Nisipeanu P Rabey JM Orlov E Catz T Kippervasser S Schechtman E Korczyn AD Double-blind comparison of cabergoline and bromocriptine in Parkinson's disease patients with motor fluctuations Neurology 1996 47 3 785 788 10.1212/WNL.47.3.785 8797480

[B25] Lozano AM Lang AE Galvez-Jimenez N Miyasaki J Duff J Hutchinson WD Dostrovsky JO Effect of GPi pallidotomy on motor function in Parkinson's disease Lancet 1995 346 8987 1383 1387 10.1016/S0140-6736(95)92404-3 7475819

[B26] Tsuji S [MSA update] Rinsho Shinkeigaku 2005 45 11 821 823 16447735

[B27] Carskadon MA Dement WC Normal human sleep: an overview Principles and practice of sleep medicine 2011 5 Elsevier Saunders, St. Louis 16 26

[B28] Shimohata T Tomita M Nakayama H Aizawa N Ozawa T Nishizawa M Floppy epiglottis as a contraindication of CPAP in patients with multiple system atrophy Neurology 2011 76 21 1841 1842 10.1212/WNL.0b013e31821ccd07 21606457

[B29] Uemura Y Nomura T Inoue Y Yamawaki M Yasui K Nakashima K Validation of the Parkinson's disease sleep scale in Japanese patients: a comparison study using the Pittsburgh Sleep Quality Index, the Epworth Sleepiness Scale and Polysomnography J Neurol Sci 2009 287 1–2 36 40 19804890 10.1016/j.jns.2009.09.015

[B30] Ozawa T Tada M Kakita A Onodera O Ishihara T Morita T Shimohata T Wakabayashi K Takahashi H Nishizawa M The phenotype spectrum of Japanese multiple system atrophy J Neurol Neurosurg Psychiatry 2010 81 11 1253 1255 10.1136/jnnp.2009.182576 20571046

[B31] Kapur VK Baldwin CM Resnick HE Gottlieb DJ Nieto FJ Sleepiness in patients with moderate to severe sleep-disordered breathing Sleep 2005 28 4 472 477 16171292 10.1093/sleep/28.4.472

[B32] Suzuki K Miyamoto M Miyamoto T Iwanami M Hirata K Sleep disturbances associated with Parkinson's disease Parkinsons Dis 2011 2011 219056 21876839 10.4061/2011/219056 PMC3159123

